# Factor VIII Intron 22 Inversion in Severe Hemophilia A Patients in Palestine

**DOI:** 10.1155/2020/3428648

**Published:** 2020-09-25

**Authors:** Caesar Mahmoud Abu Arra, Fekri Samarah, Nael Sudqi Abu Hasan

**Affiliations:** ^1^Medicare Labs, Tulkarm, State of Palestine; ^2^Department of Medical Laboratory Sciences, Arab American University (AAUP), Jenin, State of Palestine; ^3^Department of Biology and Biotechnology, An-Najah National University, Nablus, State of Palestine

## Abstract

**Background:**

Hemophilia A is an X-linked recessive bleeding disorder caused by mutations in FVIII gene with an incidence of 1 in 5,000 to 10,000 live born males. The Inv22 mutation is a major cause of the disease worldwide, accounting for up to 40%–50% of severe FVIII mutations. The aim of the present study was to screen Inv22 of the FVIII gene in Palestinian patients with severe HA and reveal its role as a predisposing factor for the development of inhibitors.

**Materials and Methods:**

A cohort of 77 HA individuals including 5 carrier females from 52 unrelated families registered at governmental hemophilia centers in the West Bank area of Palestine was investigated. The demographic data and the clinical history were retrieved from medical files. Molecular analysis of Inv22 mutation in severe HA (30 cases) from Palestine was performed using the subcycling polymerase reaction (S-PCR). FVIII coagulant activities were carried out on an aPTT-based 1-stage clotting assay. FVIII inhibitors were quantified using the Nijmegen modification of the Bethesda assay.

**Result:**

Overall, 41.7% (30/72) of the studied cases were classified as having severe HA, 22.2% (16/72) had moderate HA, and 36.1% (26/72) had mild HA. Five randomly selected carrier mothers were screened for the Inv22 mutation to confirm its transmission to their sons. The Inv22 mutation was detected in 11 severe HA patients (36.6%). Among the severe HA patients with positive Inv22, 45.5% (5/11) had developed inhibitors. The current study showed that there was no association (*p*=0.53) between inhibitor development and the Inv22 mutation.

**Conclusion:**

Findings on Inv22 are in agreement with worldwide reports, being a major genetic mutation in severe HA. The S-PCR is a simple, rapid, and cost-effective method for the diagnosis of Inv22 in severe HA patients. Although the Inv22 mutation was associated with 36.6% of severe HA phenotype cases, it was not a major predisposing factor for inhibitor formation.

## 1. Introduction

Hemophilia A (HA) is an X-linked recessive bleeding disorder and the second most common coagulation disorder with an incidence of 1 in 5,000 to 10,000 live born males [1]. HA results from a wide spectrum of heterogeneous mutations in the factor VIII (FVIII) gene leading to quantitative and qualitative defects in FVIII activity, an essential cofactor in the factor X activation complex [2]. More than 900 mutations within the FVIII coding and untranslated regions have been identified [3]. Overall, nonsense, missense, splice site, frameshift mutations, and gross (>50 bp) deletions variants are responsible for almost 50% of all cases of severe HA. Admitting the molecular pathology of nonsense, gross deletions, and frameshift mutations is evident, the functional defect of amino acids mutated by a missense single nucleotide polymorphism (SNP) is not fully understood [4]. Analysis of molecular defect based on the crystal structure of FVIIIa has been used widely to elucidate the structure-function relationship of the FVIII molecule [5, 6]. Intron 22, which is caused by recombination between a sequence within intron 22 of the FVIII gene and one of the two homologous regions telomeric to the gene, and intron 1 inversions are the most frequent molecular alterations found in severe HA patients (FVIII:C <1%) with a frequency of 45–50% and 0.5–5%, respectively [7]. Moderate (FVIII:C 1–5%) and mild (FVIII:C 5–30%) phenotypes usually result from missense substitutions [8]. Different approaches, ranging from linkage analysis to mutation screening methods that identify regions of the gene as target regions for sequencing and direct sequencing of the entire gene, have been practiced for mutation detection in HA [9]. Direct sequencing is still not widely applicable because of the high cost involved [10]. A thorough FVIII mutation database and sequence resource site (HAMSTeRS) is accessible at http://europium.csc.mrc.ac.uk. In Palestine, thus far, no molecular genotyping services for HA are available. Screening for the molecular defects has become a crucial tool in hemophilia care with respect to prediction of the clinical course and safe genetic counseling of relatives. Therefore, the aim of the present study was to screen Inv22 of the FVIII gene in Palestinian patients with severe HA and reveal its role as a predisposing factor for the development of inhibitors.

## 2. Materials and Methods

### 2.1. Patient Data and Sample Collection and Storage

This was a multicenter cohort study. HA patients attending governmental hemophilia centers from three major governmental hospitals in Nablus, Jenin, and Tulkarm in West Bank region were included in the study. A total of 72 male patients with severe hemophilia A and 5 females (mothers) from 52 unrelated families were included. HA diagnosis was determined according to each patient's personal and family history of bleeding, a prolonged activated partial thromboplastin time (aPTT), and a reduced FVIII level. Clinical data such as age, gender, age at first exposure to FVIII, family history of inhibitors, and number of exposure days (EDs) were obtained from the patients using a specially designed questionnaire. Approval for the study was obtained from the Board or Ethics Committee at An-Najah National University, Nablus, Palestine. All of the patients or their guardians for minors provided informed consent for the molecular studies. British Committee for Standards in Hematology Guidelines (http://bcshguidelines.org) was followed for the patients' selection criteria. Based on their conformation to the analogous FVIII level, 41.7% (30/72) of the studied cases were classified as having severe HA, 22.2% (16/72) had moderate HA, and 36.1% (26/72) had mild HA. All patients were tested for factor VIII activities that were carried out on a 1-stage aPTT-based clotting assay via the Stago blood coagulation analyzer (STA Compact Max; Diagnostica Stago, Asnières-sur-Seine, France) according to the manufacturer's instructions. 10 ml of peripheral blood samples was collected in two tubes: tripotassium ethylene-diamine-tetraacetate (EDTA K3) and 3.2% trisodium citrate tubes (Greiner Bio-One, Austria). Blood samples were taken at least 1 week following FVIII infusion. EDTA K3 tubes were stored at 4°C and were used later on for genomic DNA extraction. The 3.2% trisodium citrate tubes were processed immediately and centrifuged at 5000 rpm for 10 minutes, and the plasma was aspirated and stored at −20°C and used later on for FVIII activity measurements assay and the presence of inhibitors.

The aPTT mixing study was carried out to detect the presence of inhibitors. Out of the 72 male patients, 17 (23.6%) developed inhibitors: 14 patients had severe and 3 patients had moderate disease. Inhibitor titrations were carried out using the Nijmegen modification of the Bethesda assay. The majority of patients manifested high titers and 3 patients showed low titers. The patients showed a mean antibody titer of 93.6 BU/mL (range: 7–795 BU/mL).

### 2.2. DNA Extraction

DNA was extracted from 200 *μ*L of buffy coat using the Qiagen DNA Blood Mini kit (Qiagen, Hilden, Germany), following manufacturer's recommendation. DNA concentration and purity were determined in a Nanodrop 2000 spectrophotometer (Nanodrop, Wilmington, DE).

### 2.3. Subcycling PCR

The primer sequences used for the Inv22 PCR reactions (P, Q, A, and B) are shown in Table 1. PCR primer sequences are indicated from 5′ to 3′ end with an additional nucleotide to serve as a GC-clamp [11, 12]. Primer sequences were checked for specificity using NCBI Primer-BLAST and UCSC In-Silico PCR (http://www.genome.ucsc.edu). The primers were used to differentiate between normal, inversion patients and carrier females. Primers P & Q are specific to flanking sequences of int22h1 located at −1212 bp before and at +1334 bp after the homolog, and produce 12 kb segment in DNA of wild type and carrier females. Primers A & B are specific to the flanking sequences of int22h2 and int22h3 located at −167 bp before and at +118 bp after the homologs and always produce 10 kb segment, because at least one copy of Int22h2 and Int22h3 remains intact and could serve as a positive control. Inv22 can occur by homologous recombination between Int22h1 and either Int22h2 or Int22h3; thus, each P & B and A & Q produces 11 kb segments in DNA of male patients and carrier females. S-PCR reaction was performed using Expand Long Template PCR kit (Roche, Mannheim, Germany) as previously described with minor modification of the original protocol [11–13]. Four combinations of two primers A & B, P & Q, A & Q, and P & B per sample were performed for detection of Inv22 in the FVIII gene, which was performed in four single reactions for severe HA patients as well as for expected carrier mothers' samples. Bio-Rad Thermal Cycler (T100™, Foster City, California, USA) was used for DNA amplification. The PCR cycling conditions were as follows: initial denaturation at 95°C for 2 minutes; the first 10 cycles at 95°C for 30 seconds, followed by four subcycles of annealing/elongation step alternating temperature between 63°C and 68°C for 2 minutes for each subcycle. The remaining 20 cycles were modified by the addition of an extra 3 seconds per cycle for each step of the annealing/elongation step, followed by a final elongation step of 68°C for 7 minutes. PCR was performed in a total volume of 25 *μ*l containing 2.5 *μ*l buffer, 0.75 mM MgCl_2_, 1.75 U enzyme mix, 200 *μ* mol 7-deaza-dGTP (Roche, Mannheim, Germany), 300 *μ* mol GTP and 500 *μ* mol of each of the other dNTPs (Sigma, Deisenhofen, Germany), 0.2 *μ* mol of each forward and reverse primer (hylabs, Ltd., Israel), 7.5% dimethyl sulphoxide (DMSO), and 50–100 ng of human genomic DNA template. Amplified products were resolved in 1% agarose (Sigma, Deisenhofen, Germany) in 1x TBA buffer (Promega, Madison, USA) and visualized using ultraviolet transilluminator documentation system (Uvitec, Cambridge, UK).

## 3. Results

In this study, 72 male patients with hemophilia A and 5 carrier mothers from 52 unrelated families aged 1 month to 67 years (mean: 22.5 ± 15.4 years, median: 19 years) were investigated. All patients tested for Inv22 were varying with respect to family history, treatment regimen, first bleeding site, and age at first bleeding. Family history of bleeding was present in 96.7%. Age at first bleeding was from less than 1 month to 2 years. First bleeding was reported in 83.3% at circumcision, 6.7% after trauma, and 10% due to muscle hematoma. According to treatment regimen, all individuals were treated with FVIII concentrate (plasma derived), and 76.7% of them received on-demand treatment, while the rest 23.3% were on prophylactic treatment (Table 2).

The Inv22 mutation was detected in 11 patients (36.6%), and all of them had severe HA.

Among the severe HA patients with positive Inv22, 45.5% (5/11) developed inhibitors.  Table 3 summarizes the characteristics of severe HA patients with regard to age, age at first exposure to FVIII concentrates, exposure days, family history of inhibitors, and inhibitors production with respect to Inv22. The current study showed that there was no association (*p*=0.53) between inhibitor development and the Inv22 mutation.

**Figure 1:**
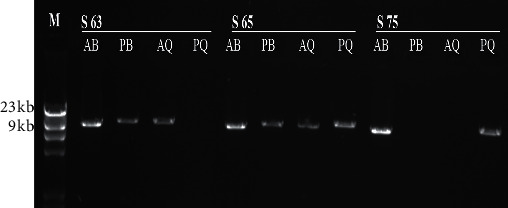
S-PCR analyses for FVIII intron 22 related inversion using 1% agarose gels. AB, PB, AQ, and PQ are primers related bands for samples 63, 65, and 75. The picture shows an Inv22 positive result for sample S63, a heterozygous carrier female Inv22 for sample S65, and a negative Inv22 result for sample 75. M: *λ* DNA HindIII ladder marker (0.1 kb–23 kb). S: sample number.

**Figure 2:**
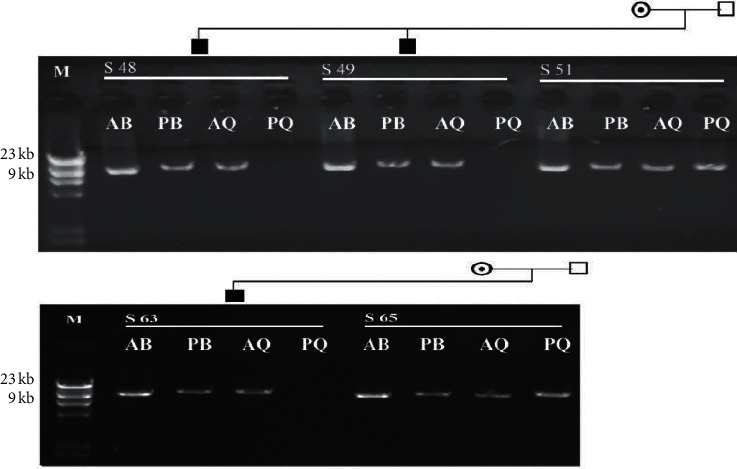
Family pedigree analysis of the carrier females included in the current study. AB, PB, AQ, and PQ are primers related bands. ʘ means carrier; ■ means affected male; □ means unaffected male. M: *λ* DNA HindIII marker (0.1 kb–23 kb); S: sample number.

Mutational analysis using S-PCR technique was carried out for all 30 HA severe cases. Analysis also included 5 randomly selected mothers from these patients, for evaluation of carrier status of Inv22, and 15 wild type healthy male controls. Affected males showed positive amplification for three bands, AB, PB, and AQ, and absence of PQ amplified products (12 kb segment). Analysis showed that, out of the 30 tested severe hemophilia A cases, 11 (36.6%) were positive for Inv22. A representative gel photograph for S-PCR analysis is shown in Figure 1. From the 5 randomly selected female carriers, only 2 showed positive amplifications of all bands, AB, PB, AQ, and PQ, indicating the presence of Inv22 in these females. Family pedigree analysis for these 2 female carries, shown in Figure 2, emphasizes the role of transmission of this inversion from carrier mothers to their sons.

## 4. Discussion

To the best of our knowledge, this is the first study to describe the health status of HA patients in the West Bank area of Palestine and to assess the prevalence of Inv22 mutation in X chromosome among severe HA patients as well as suspected carriers, being a major causative of this disease worldwide. Based on clinical data of early symptoms presentation and family history, one can easily predict the severity of the disease in the area. Since family history is strongly correlated with severity of disease, early screening diagnosis for families at risk, performing aPTT before circumcision will be helpful and cost effective. Early diagnosis and adequate and timely prophylaxis treatment can prevent complications like hemarthrosis and life-threatening bleedings. They are also expected to improve the quality of life and decrease the morbidity and mortality rates [14]. The aim of the present study was to screen Inv22 of the FVIII gene in Palestinian patients with severe HA and reveal its role as a predisposing factor for the development of inhibitors. Findings on factor VIII activity test showed that 41.7% of the studied cases had severe hemophilia. These findings ensure the need for adopting early screening strategy for new borne infants of families at risk in our area. Unfortunately, no sufficient data is available for HA complications such as FVIII inhibitor development, musculoskeletal complications, anemia, and infectious diseases, which makes us unable to correlate these complications with the severity of the disease. It is also worth noting that most of hemophiliac A individuals in Palestine have limited access to FVIII concentrate and are treated on demand instead of prophylactic treatment, which increases the risk of infections and disease complications among these patients. The Inv22 arises almost entirely in males due to the unpaired X chromosome during spermatozoa meiosis and is mediated by an intrachromosomal homologous recombination between a region of 9.5 kb within FVIII intron 22 (int22h1) and 1 of 2 inversely oriented copies of this sequence, int22h2 and int22h3, also known as proximal and distal homologs, respectively. Inv22 mutation is responsible for severe HA disease in about 45–50% of individuals worldwide [7]; therefore, it is reasonable to consider it as the first line for testing among these patients. The prevalence of Inv22 mutation among severe HA patients in Palestine was 36.7% based on the findings of S-PCR analysis. This frequency is consistent with previous reports from Iraq (36.3%), Japan (33.3%), Brazil (39.4%), and China (37%) and lower than frequency reported in Jordan (52%), Saudi Arabia (50%), Egypt (46.1%), and Iran (47%), but higher than that reported for Lebanon (29%), Tunis (22.7%), United Kingdom (17.6%), and Albania (10.5%) [3, 4, 15–17]. Differences in reported prevalence rates from different countries were attributed in some studies to the limited number of studied cases, the ethnic variations, and the inclusion of patients with moderate and mild FVIII activity results. In the absence of Inv22 mutation, other genetic testing should be considered, in order to elucidate the causes of the disease among severe hemophilia A patients in our area, including Inv1 mutation on the same chromosome and full gene sequencing. In our study, 45.5% (5/11) of patients with Inv22 were found to have inhibitors. According to data obtained from previously published studies, the prevalence of inhibitors in patients with intron 22 inversions was quite variable [18–21]. Astermark et al. and Goodeve et al. have reported that the type of FVIII gene mutation, particularly Inv22, is a major genetic factor contributing to inhibitor formation in severe HA patients [22, 23]. The present study showed that in Palestine, as in other countries, intron 22 inversion is probably the most common mutation in severe HA and is a major genetic factor involved in inhibitor formation [18–23]. Our study showed that there was no association (*p*=0.53) between inhibitor development and the Inv22 mutation.

According to treatment regimen in Palestinian Ministry of Health (MOH), all HA patients are treated with FVIII concentrate (plasma derived). In this study, 76.7% of HA patients received on-demand treatment. Ghosh et al. [24] reported a much lower prevalence rate of inhibitors (8.2%) in patients with severe HA in India who, like Palestinian patients, were treated on demand. Ghosh et al. suggested that occasional treatment of patients allows for the disappearance of the inhibitors from the circulation between treatment episodes, thus lowering the prevalence rate, particularly for transient inhibitors [24].

Since hemophilia is an X-linked disorder, it was reasonable to carry out pedigree analysis involving expected carrier female cases reported in this study. Data on S-PCR analysis, involving 2 expected carrier mothers of severe HA cases and presented in Figure 2, clearly showed the mode of transmission X-linked mutations from mothers to sons as both mothers in the pedigree transmitted Inv22 mutation to their sons.

## 5. Conclusion

We report here the first molecular analysis of Inv22 mutation as a major genetic mutation responsible for severe HA patients in Palestine. Our results are consistent with the reports in neighboring countries and countries all over the world. The S-PCR is a simple, rapid, and cost effective method for diagnosis of Inv22 in severe HA patients. Although the Inv22 mutation was associated with 36.6% of severe HA phenotype cases, it was not a major predisposing factor for inhibitor formation. Further studies are recommended with large number of patients as well as screening for other mutations such as Inv1 and full genome sequencing for novel mutations among negative Inv22 patients.

## Figures and Tables

**Table 1:** PCR primer sequences for amplification of factor VIII Inv22.

Primer	Sequence (5′ ⟶ 3′)	Length	GenBank accession no.	Specificity	Product sizes
P	GCC CTG CCT GTC CAT TAC ACT GAT GAC ATT ATG CTG AC	38	AF062514	Forward for int22h1	AB = 10 kb WT, carrier, inversion
Q	GGC CCT ACA ACC ATT CTG CCT TTC ACT TTC AGT GCA ATA	39	X86012	Reverse for int22h1	PQ = 12 kb WT, carrier
A	CAC AAG GGG GAA GAG TGT GAG GGT GTG GGA TAA GAA	36	AF062515	Forward for int22h2 & 3	PB = 11 kb carrier, inversion
B	CCC CAA ACT ATA ACC AGC ACC TTG AAC TTC CCC TCT CAT A	40	AF062516	Reverse for int22h2 & 3	AQ = 11 kb carrier, inversion

**Table 2:** Clinical and demographic data of severe HA patients from Palestine.

ID	Gender/age in years	Age at first bleeding	Clinical severity	Treatment regimen	Family history	Inv22
S 1	M/44	2 years	Severe	Prophylaxis	Yes	Yes
S 3	M/6	<1 month	Severe	On demand	Yes	No
S 6	M/18	2 years	Severe	On demand	Yes	No
S 7	M/12	<1 month	Severe	On demand	Yes	Yes
S 8	M/9	<1 month	Severe	On demand	Yes	Yes
S 10	M/36	3 months	Severe	On demand	Yes	No
S 16	M/15	<1 month	Severe	Prophylaxis	Yes	No
S 18	M/9	<1 month	Severe	On demand	Yes	No
S 19	M/22	<1 month	Severe	On demand	Yes	No
S 20	M/30	<1 month	Severe	On demand	Yes	No
S 22	M/15	<1 month	Severe	On demand	Yes	No
S 27	M/48	<1 month	Severe	Prophylaxis	Yes	Yes
S 30	M/16	<1 month	Severe	On demand	Yes	No
S 33	M/5	6 months	Severe	Prophylaxis	Yes	No
S 35	M/17	<1 month	Severe	On demand	Yes	Yes
S 36	M/14	<1 month	Severe	On demand	Yes	No
S 37	M/40	<1 month	Severe	On demand	Yes	No
S 43	M/6	<1 month	Severe	Prophylaxis	Yes	Yes
S 48	M/6	6 months	Severe	On demand	Yes	Yes
S 49	M/0.1	<1 month	Severe	On demand	Yes	Yes
S 52	M/5	<1 month	Severe	On demand	Yes	No
S 55	M/3	<1 month	Severe	Prophylaxis	Yes	No
S 56	M/11	<1 month	Severe	Prophylaxis	Yes	No
S 59	M/24	<1 month	Severe	On demand	Yes	No
S 61	M/10	<1 month	Severe	On demand	Yes	Yes
S 62	M/14	<1 month	Severe	On demand	Yes	Yes
S 63	M/3	<1 month	Severe	On demand	Yes	Yes
S75	M/4	<1 month	Severe	On demand	Yes	No
S 76	M/2	<1 month	Severe	On demand	No	No
S 77	M/6	<1 month	Severe	On demand	Yes	No
S 17	F/35				Yes	No
S 53	F/29				Yes	No
S 57	F/30				Yes	No
S 51	F/37				Yes	Carrier
S 65	F/25				Yes	Carrier

**Table 3:** Severe HA patients' studied characteristics with regard to age, age at first exposure to FVIII concentrates, exposure days, family history of inhibitors, and inhibitors production with respect to Inv22.

Studied characteristics	Severe HA cases (*N* = 30)	
	Positive for inhibitors (*N* = 14)	Negative for inhibitors (*N* = 16)
Mean age (years) ± SD^*∗*^, range	14 ± 6, 4–27	24 ± 7, 6–48

Age at first exposure to FVIII (*N* %)		
<6 months	7 (50%)	6 (37.5%)
≥6 months	7 (50%)	10 (62.5%)
Mean in months ± SD	16 ± 12	29 ± 38

Exposure days (*N* %)		
<120	14 (100%)	5 (31.3)
≥120	0 (0%)	11 (68.7%)
Mean days of exposure ± SD	64 ± 21	152 ± 73
Range	6–120	28–377

Family history of inhibitors (*N* %)	13 (92.9%)	3 (11.5%)

Inv22 mutation with inhibitors (*N* %)		
Positive for Inv22	5 (45.5%)	6 (54.5%)
Negative for Inv22	9 (47.4%)	10 (52.6%)
Total	14 (46.7%)	16 (53.3%)

^*∗*^SD: standard deviation.

## Data Availability

The datasets used or/and analyzed during the current study are available from the corresponding author on reasonable request.
